# Effect of enzyme substitution therapy on brain magnetic resonance imaging and cognition in adults with phenylketonuria: A case series of three patients

**DOI:** 10.1111/ene.16508

**Published:** 2024-10-04

**Authors:** Alessandro P. Burlina, Renzo Manara, Jessica Carretta, Chiara Cazzorla, Christian Loro, Vincenza Gragnaniello, Alberto B. Burlina

**Affiliations:** ^1^ Neurology Unit St. Bassiano Hospital Bassano del Grappa Italy; ^2^ Neuroradiology Unit University Hospital of Padua Padua Italy; ^3^ Division of Inherited Metabolic Diseases University Hospital of Padua Padua Italy

**Keywords:** brain MRI, cognition, enzyme substitution therapy, phenylketonuria

## Abstract

Phenylketonuria, the most common inherited metabolic disease, results from a deficiency of phenylalanine hydroxylase enzyme activity that causes high blood phenylalanine levels. Most adults do not adhere to the gold standard therapy: lifelong treatment with a low‐phenylalanine diet. Elevated and fluctuating phenylalanine levels in untreated adults can cause white matter abnormalities, neurological symptoms, and cognitive dysfunction (executive function). Pegvaliase, a derivative of the phenylalanine ammonia‐lyase enzyme, metabolizes phenylalanine to trans‐cinnamic acid and ammonia, and is approved by the US Food and Drug Administration and European Medicines Agency for subcutaneous administration in adults with phenylketonuria and blood phenylalanine concentrations > 600 μmol/L. In clinical trials, it reduced blood phenylalanine, even in patients consuming an unrestricted diet. We report longitudinal results on the first three such adults, in whom phenylalanine levels were quantified monthly, starting 1 year before pegvaliase administration and continuing through achievement of a pegvaliase response (defined as six consecutive monthly blood phenylalanine concentrations < 360 μmol/L while consuming an unrestricted diet). Brain magnetic resonance imaging (MRI) and neuropsychological assessments were performed before starting therapy and after response was achieved. Our results show that all three patients had significantly reduced white matter hyperintensities on brain MRI and improved executive function on neuropsychological assessment, especially on the Paced Auditory Serial Addition Test, which is known to be very sensitive to white matter functioning. To the best of our knowledge, this is the first report of concomitant improvements in cognitive performance and white matter damage after a pharmacological intervention to normalize phenylalanine levels in adults with phenylketonuria consuming an unrestricted diet.

## INTRODUCTION

Phenylketonuria (PKU; Online Mendelian Inheritance in Man 261600) is a genetic disorder caused by a deficiency of phenylalanine (Phe) hydroxylase enzyme activity that leads to high blood Phe with cognitive dysfunction and neurological sequelae if not diagnosed and treated at birth [1, 2]. The gold standard therapy—a strict low‐Phe diet from birth—is recommended throughout life; however, adherence may decrease sharply in adolescence and adulthood, because of difficulty following the burdensome diet regimen. Consequently, harmful elevation and fluctuation of Phe levels are detected frequently [2]. Varying degrees of cognitive impairment have been reported, mainly in adults with high Phe levels [1, 3]. In many adult patients, white matter abnormalities, detected by brain magnetic resonance imaging (MRI), have been reported [1, 4].

## METHODS

We treated three adults with classical PKU. Patient 1, a 36‐year‐old male, was compound heterozygous for the variants IVS12 + 1G > A + p.Arg408Trp on the *PAH* gene. Patient 2, a 37‐year‐old female, carried the variants IVS12 + 1G > A + p.Pro281Leu. Finally, Patient 3, a 24‐year‐old female, carried the variant p.Arg408Trp in compound heterozygosis with a large deletion of exon 3. All patients had discontinued diet treatment (mean Phe levels > 1300 μmol/L; recommended safe range < 360 μmol/L) and were treated with pegvaliase, a recombinant Phe ammonia‐lyase enzyme substitution therapy that metabolizes Phe to nontoxic trans‐cinnamic acid and ammonia [5]. Doses were titrated gradually based on tolerability until the individually effective dose was reached. Maintenance dose and frequency were then adjusted to achieve Phe levels of <360 μmol/L. Phe was measured by tandem mass spectrometry in dried blood spots.

Neurological examinations, brain MRI, and neuropsychological assessments were performed before starting pegvaliase therapy, and again after Phe levels had stabilized at <360 μmol/L for 6 months (Table S1). Neuropsychological evaluations comprised 13 instruments, including the Montreal Cognitive Assessment, six instruments to measure executive functions (including information processing speed tests), two for visual attention, three for memory, and one for social cognition [6]. The neuropsychological assessment was performed during a 1.5‐ to 2‐h session. Parallel tests were used to assess long‐term memory (Table S2). Patients also underwent brain MRI scans before and after Phe stabilization. All images were acquired with the same Philips Ingenia 3‐T scanner using a 32‐channel quadrature head coil. The protocol included three‐dimensional fluid‐attenuated inversion recovery (FLAIR; repetition time [TR]/inversion time/echo time [TE] = 8000/2400/360 ms, echo train length = 180, voxel size = 1.12 × 1.12 × 1.12 mm, field of view = 247 mm) and diffusion‐weighted imaging (DWI; TR/TE = 4209/108 ms, voxel size = 1.8 × 18 mm, slice thickness = 4 mm, *b*‐values = 0 and 1500 s/mm^2^); apparent diffusion coefficient (ADC) maps were automatically generated by the instrument.

## RESULTS

After dose titration, Patients 1 and 3 received a maintenance dosage of 20 mg/day, and Patient 2 received 40 mg/day. Despite some initial injection site reactions, all patients were adherent to therapy and maintained stable Phe levels of <360 μmol/L for ≥6 months while consuming an unrestricted diet (Table S1, Figure S1).

Before treatment, the neurological examination of Patients 2 and 3 was normal, whereas that of Patient 1 showed hyperreflexia. All neurological examinations after treatment were normal.

**FIGURE 1:**
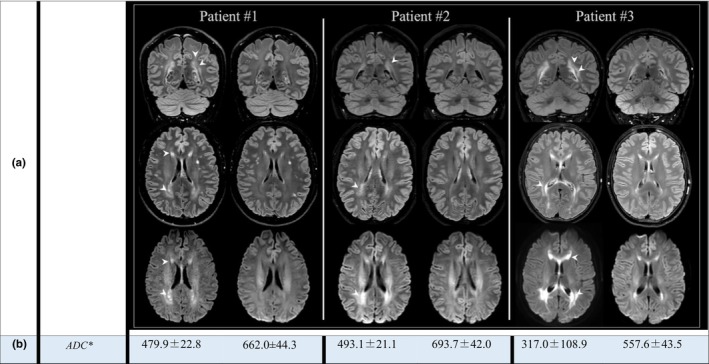
Brain magnetic resonance imaging coronal and axial fluid‐attenuated inversion recovery images and axial diffusion‐weighted images (a) and apparent diffusion coefficient (ADC) values (b) of three adult patients with phenylketonuria before treatment with pegvaliase (left column) and after phenylalanine stabilization (right column). Arrowheads indicate white matter abnormalities. *ADC values (mm^2^/s × 10^−6^); normal white matter ADC values are approximately 700 mm^2^/s × 10^−6^.

Brain MRI after Phe stabilization for ≥6 months revealed a reduction in white matter abnormalities on FLAIR and DWI, with near normalization of ADC values, a biomarker of myelin integrity (Figure 1). Neuropsychological assessments after Phe stabilization showed improvements in information processing speed. Although our cohort for analysis included only three patients, all showed a consistent improvement in Paced Auditory Serial Addition Test (PASAT) scores (a mean percentage of 34%); Patient 2 reached the maximum score of 60, starting from 45.4 (normal value = 30–60; Figure S2, Table S2). For all three patients, no significant changes have been reported for the Symbol Digit Modalities Test.

## DISCUSSION

We confirm previous reports that pegvaliase can achieve and maintain Phe levels of <360 μmol/L [2, 5], while simultaneously allowing patients to increase their intake of natural protein (Figure S1). Our results show that maintaining Phe levels in the safe range was associated with a significant reduction in white matter abnormalities (FLAIR and ADC values), and a parallel improvement in information processing speed, as detected by PASAT, a specific test for assessing white matter function (Figure 1, PASAT score in the Table S1, Figure S2) [7].

In a double‐blind, randomized, placebo‐controlled crossover trial, Muri et al. found that 4‐week exposure to high Phe in adults with PKU reduced cortical thickness and increased white matter volume compared to placebo [8], supporting the brain toxicity of high Phe levels in adults and confirming that prolonged high Phe levels should be avoided. These structural alterations returned to baseline measures during placebo/washout periods [8].

To the best of our knowledge, ours is the first report of concomitant improvements in cognitive performance and white matter damage after a pharmacological intervention to almost normalized Phe levels in adults with PKU consuming an unrestricted diet.

## AUTHOR CONTRIBUTIONS

**Alessandro P. Burlina:** Conceptualization; writing – original draft; writing – review and editing. **Renzo Manara:** Investigation; formal analysis; writing – review and editing. **Jessica Carretta:** Investigation; formal analysis; writing – review and editing. **Chiara Cazzorla:** Data curation; writing – review and editing. **Christian Loro:** Data curation; writing – review and editing. **Vincenza Gragnaniello:** Data curation; writing – review and editing. **Alberto B. Burlina:** Conceptualization; writing – review and editing.

## CONFLICT OF INTEREST STATEMENT

The authors declare no conflict of interest.

## ETHICS STATEMENT

This study was approved by the Research Ethics Committee of the University Hospital of Padua (no. 8260, 02/02/2024). All patients provided informed consent for participation in the study, conducted in accordance with the principles of the Declaration of Helsinki.

## Supporting information


Data S1

## Data Availability

The data that support the findings of this study are available on request from the corresponding author. The data are not publicly available due to privacy or ethical restrictions.
