# Supplementary material for: Effect of enzyme substitution therapy on brain magnetic resonance imaging and cognition in adults with phenylketonuria: A case series of three patients
Source: Eur J Neurol. 2024 Oct 4;31(12):e16508. doi: 10.1111/ene.16508 (PMC11554989; doi:10.1111/ene.16508)
Supplement: Supplementary file 1 — Data S1 [file ENE-31-e16508-s001.docx]

**Supplemental Material**

**
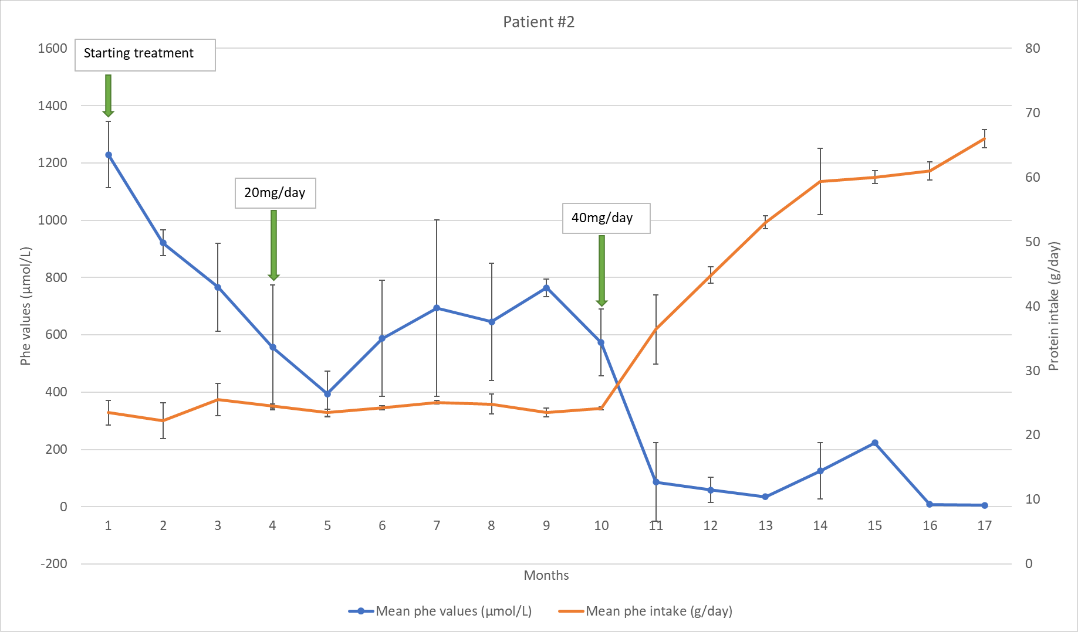
Supplemental Figure e1:** Blood phenylalanine levels (mean ± SD) and daily protein intake of the three adults with PKU consuming a free diet (mean ± SD)


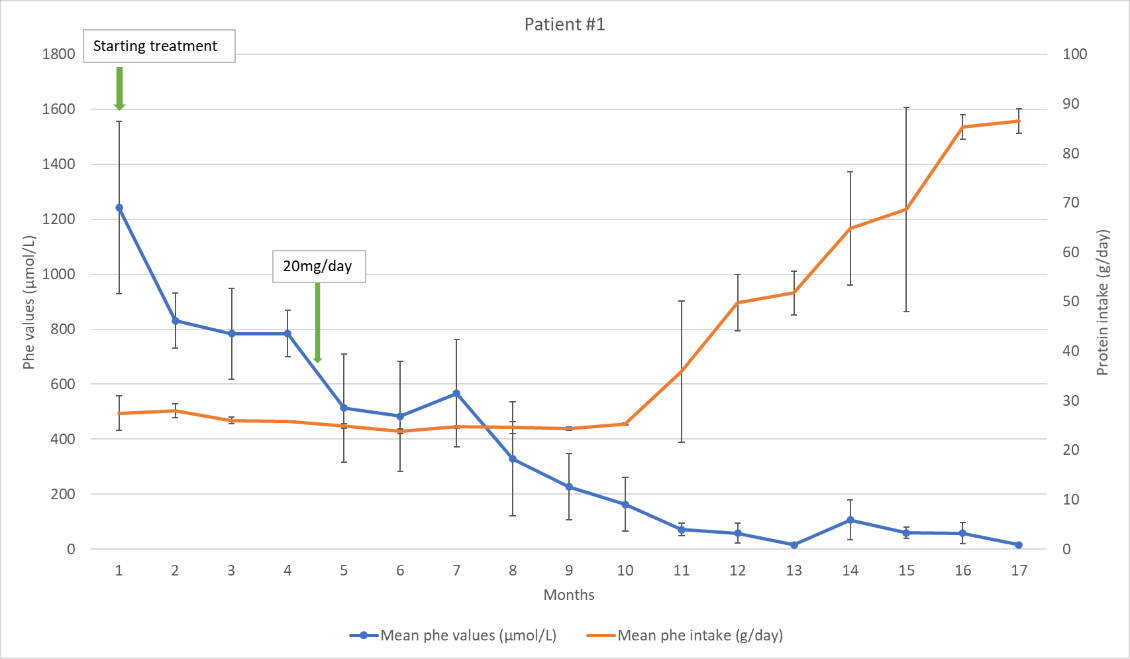


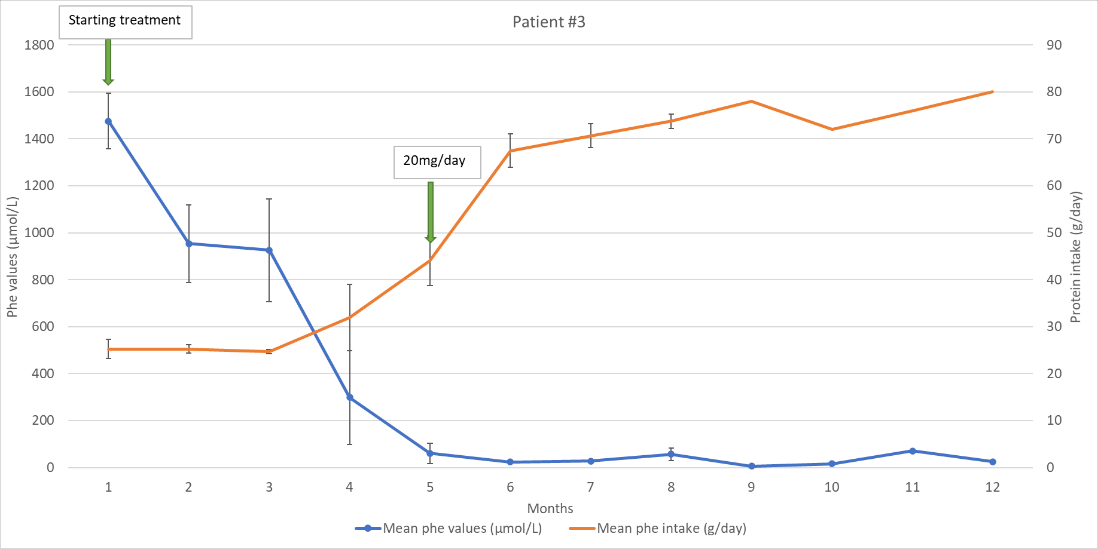


**Supplemental Figure e2:** Dot plot of PASAT scores of PKU patients. Each dot represents the mean value (n=3) of the PASAT scores before treatment (BT) and after treatment (AT); p<0.025


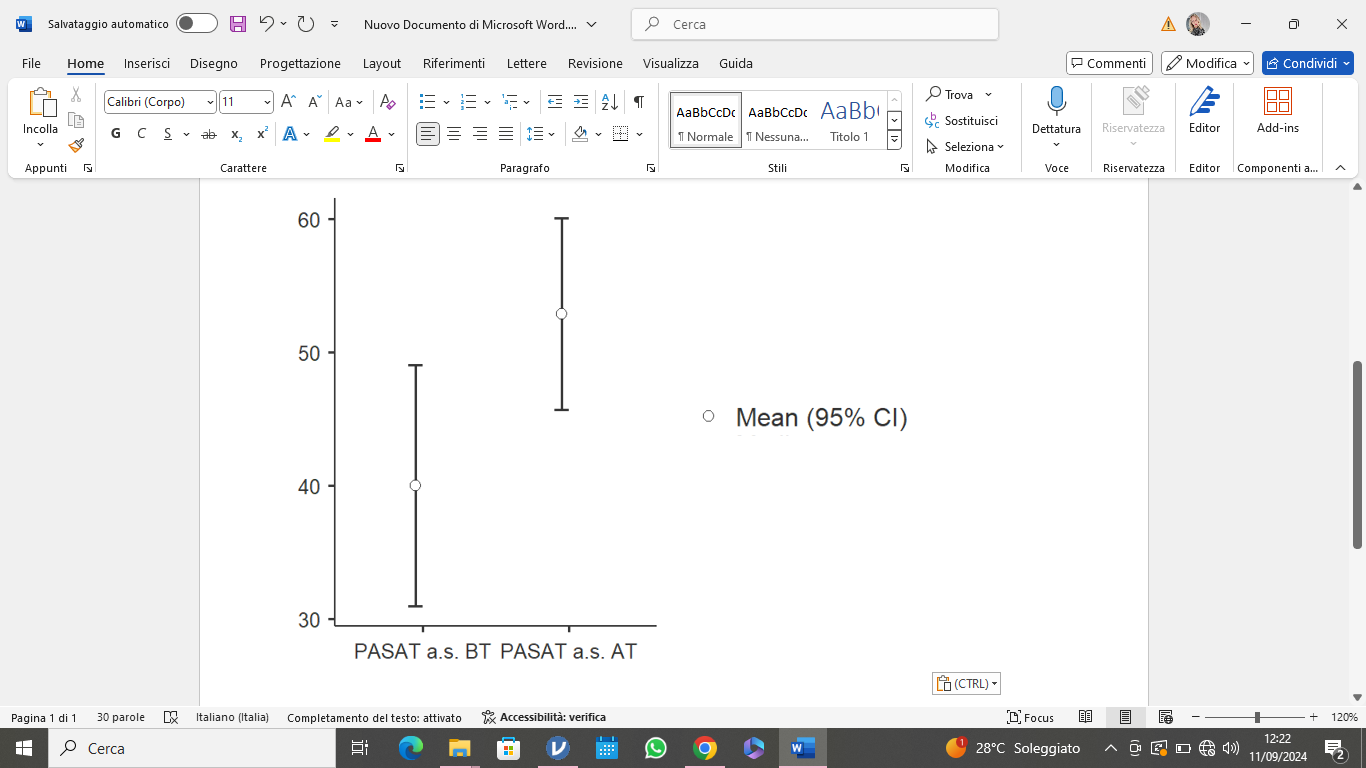


**Supplemental Table e1:** Demographic and clinical data of the three adults with PKU before and after achieving stable blood phenylalanine levels < 360 μmol/L for at least 6 months.

|  | | | **Patient 1** | | **Patient 2** | **Patient 3** |
| --- | --- | --- | --- | --- | --- | --- |
| **Baseline characteristics** | | | | | | |
| Age at enrollment (years) | | | 36 | | 37 | 24 |
| Sex | | | M | | F | F |
| Ethnicity | | | Latin | | Latin | Latin |
| Diagnosis at neonatal screening | | | + | | + | + |
| PAH protein variants | | | IVS12+1G>A + p.Arg408Trp | | IVS12+1G>A + p.Pro281Leu | p.Arg408Trp + deletion exon 3 |
| Weight (kg) | | | 66 | | 64 | 60 |
| Height (cm) | | | 170 | | 157 | 162 |
| BMI (kg/m^2^) | | | 23 | | 26 | 23 |
| **PKU treatment** | | | Medical food alone | | Medical food alone | Medical food alone |
| Daily protein intake from medical foods (mean, g) | | | 63 | | 64 | 56 |
| Daily protein intake from natural foods (mean, g) | | | 29 | | 25 | 25 |
| Daily Phe intake (mean, mg) | | | 1450 | | 1250 | 1250 |
| Phe levels (last 3 years) | Mean (µmol/l) | | 1314 | | 1343 | 1453 |
|  | Range (µmol/l) | | 913-1642 | | 955-1857 | 1042-1667 |
|  | Number of samples* | | 34 | | 65 | 48 |
| Last Phe pre-treatment µmol/l | | | 1242 | | 1358 | 1567 |
| Diet adherence | | | poor | | poor | poor |
| Education | | | Secondary high school | | Postgraduate degree | Secondary high school |
| Employment status | | | Employed | | Employed | Employed |
| Neurological examination | | | Hyperreflexia | | Normal | Normal |
| Cognitive assessment (PASAT) ** | | | 45.4/60 | | 43.78/60 | 30.79/60 |
| Brain MRI | | | White matter abnormalities | | White matter abnormalities | White matter abnormalities |
| **Receiving subcutaneous pegvaliase treatment** | | | | | | |
| Age at assessment (years) | | | 38 | | 39 | 25 |
| Treatment duration (months) | | | 17 | | 17 | 12 |
| Daily maintenance dosage (mg) | | | 20 | | 40 | 20 |
| Time on maintenance (months) | | | 9 | | 7 | 7 |
| Daily protein intake from natural foods (mean, g) | | | 88 | | 65 | 80 |
| Phe levels (last 6 months) | Mean (µmol/l) | | 48 | | 168 | 64 |
|  | Range (µmol/l) | | 8-140 | | 7-235 | 21-184 |
|  | Number of samples* | | 35 | | 22 | 19 |
| Neurological examination | | Normal | | Normal | | Normal |
| Cognitive assessment (PASAT) ** | | 60/60 | | 50.78/60 | | 47.79/60 |
| Brain MRI | | Reduction of white matter abnormalities | | Reduction of white matter abnormalities | | Reduction of white matter abnormalities |

Abbreviations: BMI: body mass index; MRI: magnetic resonance imaging. *dried blood spots; **PASAT (Paced Auditory Serial Addition Test) adjusted score.

| **Supplemental Table e2:** Cognitive Assessment of the three adults with PKU before and after phenylalanine stabilization with pegvaliase treatment. RS=Raw Score; AS=Adjusted Score; ES=Equivalent Score, 4=Upper Normal Level, 3=Normal, 2=Lower Normal Level, 1=Borderline, 0=Deficit | | | | | | | | | | | | | | | | | | | |
| --- | --- | --- | --- | --- | --- | --- | --- | --- | --- | --- | --- | --- | --- | --- | --- | --- | --- | --- | --- |
|  | **CUT-OFF** | **Patient #1** | | | | | | **Patient #2** | | | | | | **Patient #3** | | | | | |
|  |  | **Before treatment** | | | **After Phe stabilization** | | | **Before treatment** | | | **After Phe stabilization** | | | **Before treatment** | | | **After Phe stabilization** | | |
|  |  | **R.S.** | **A.S.** | **E.S./ Result** | **R.S.** | **A.S.** | **E.S./Result** | **R.S.** | **A.S.** | **E.S./Result** | **R.S.** | **A.S.** | **E.S./Result** | **R.S.** | **A.S.** | **E.S./Result** | **R.S.** | **A.S.** | **E.S./Result** |
| **Screening** | | | | | | | | | | | | | | | | | | | |
| The Montreal Cognitive Assessment – MoCA (0-30) ^1^ | ≤ 15.5 | 26 | 26.98 | 4 | 25 | 25.98 | 4 | 29 | 25.85 | 4 | 28 | 24.85 | 4 | 28 | 28.72 | 4 | 28 | 28.72 | 4 |
| **Visual Attention** | | | | | | | | | | | | | | | | | | | |
| Digit Cancellation Test (0-50) ^2^ | < 24 | 42 | 35.5 | 2 | 47 | 40.5 | 4 | 49 | 40.9 | 4 | 49 | 40.9 | 4 | 39 | 33.6 | 2 | 44 | 38.6 | 3 |
| Trail Making Test – TMT ^3^ |  |  |  |  |  |  |  |  |  |  |  |  |  |  |  |  |  |  |  |
| - Part A | ≥ 94 | 33 | 33 | 4 | 21 | 21 | 4 | 23 | 36 | 4 | 23 | 36 | 4 | 37 | 46 | 3 | 29 | 38 | 4 |
| - Part B | ≥ 282 | 69 | 62 | 4 | 73 | 66 | 4 | 56 | 105 | 3 | 57 | 106 | 3 | 61 | 84 | 4 | 62 | 85 | 4 |
| - Part (B-A) | ≥ 187 | 36 | 29 | 4 | 52 | 45 | 4 | 33 | 69 | 3 | 34 | 70 | 3 | 24 | 38 | 4 | 33 | 47 | 4 |
| **Memory** | | | | | | | | | | | | | | | | | | | |
| Digit Span Memory Test ^4^ |  |  |  |  |  |  |  |  |  |  |  |  |  |  |  |  |  |  |  |
| - Forward (0-8) | < 4.26 | 6 | 5.76 | 4 | 6 | 5.76 | 4 | 6 | 5.41 | 3 | 7 | 6.41 | 4 | 7 | 6.61 | 4 | 7 | 6.61 | 4 |
| - Backward (0-8) | < 2.65 | 4 | 3.84 | 3 | 4 | 3.84 | 3 | 7 | 6.31 | 4 | 6 | 5.31 | 4 | 5 | 4.69 | 4 | 4 | 3.69 | 2 |
| Corsi Span Test-Forward (0-8) ^4^ | < 3.46 | 6 | 5.80 | 4 | 4 | 3.80 | 1 | 6 | 5.37 | 3 | 5 | 4.37 | 4 | 4 | 3.65 | 1 | 8 | 8 | 4 |
| Rey Auditory Verbal Learning Test – RAVLT ^5^ |  |  |  |  |  |  |  |  |  |  |  |  |  |  |  |  |  |  |  |
| - Immediate (0-75) | ≤ 28.52 | 53 | 48.2 | 4 | 50 | 45.2 | 4 | 61 | 51.7 | 4 | 55 | 45.7 | 4 | 54 | 45.9 | 4 | 56 | 47.9 | 4 |
| - Deferred (0-15) | ≤ 4.68 | 11 | 9.3 | 4 | 11 | 9.3 | 3 | 15 | 15 | 4 | 14 | 11.2 | 4 | 13 | 10.2 | 4 | 13 | 10.2 | 4 |
| **Executive Functions** | | | | | | | | | | | | | | | | | | | |
| Stroop Test ^6^ |  |  |  |  |  |  |  |  |  |  |  |  |  |  |  |  |  |  |  |
| - Errors | ≥ 4.24 | 0 | 0 | 4 | 0 | 0 | 4 | 2 | 3.50 | 1 | 0 | 0 | 4 | 0 | 0 | 4 | 0 | 0 | 4 |
| - Time | ≥ 36.92 | 11.5 | 18 | 4 | 14.5 | 21 | 4 | 13 | 23.50 | 3 | 12.5 | 23 | 3 | 22 | 35.5 | 1 | 12 | 25.5 | 3 |
| Hayling Sentence Completion Test ^7^ |  |  |  |  |  |  |  |  |  |  |  |  |  |  |  |  |  |  |  |
| - Time Score | ≤ 5 | 87 | 1 | Deficit | 8 | 9 | Normal | 15 | 5 | Deficit | 26 | 1 | Deficit | 13.5 | 7 | Normal | 0 | 19 | Upper Normal Level |
| - Error Score | ≤ 5 | 7 | 9 | Normal | 1 | 14 | Upper Normal Level | 6 | 10 | Normal | 2 | 13 | Upper Normal Level | 6 | 9 | Normal | 3 | 12 | Upper Normal Level |
| Phonemic Verbal Fluency ^8^ | < 17.77 | 21 | 26.91 | 3 | 22 | 27.91 | 3 | 52 | 44.71 | 4 | 55 | 47.71 | 4 | 41 | 43.73 | 4 | 56 | 58.73 | 4 |
| Symbol Digit Modalities Test – Oral Version SDMT ^9^ | ≤ 34.19 | 58 | 55.3 | Normal | 54 | 51.3 | Normal | 60 | 47.9 | Normal | 62 | 49.9 | Normal | 42 | 34.9 | Normal | 46 | 38.9 | Normal |
| Paced Auditory Serial Addition Test (0-60) ^10^ | < 28.95 | **41** | **45.4** | **3** | **57** | **60** | **4** | **50** | **43.78** | **3** | **57** | **50.78** | **4** | **29** | **30.79** | **1** | **46** | **47.79** | **4** |
| Tower Of London ^11^ |  |  |  |  |  |  |  |  |  |  |  |  |  |  |  |  |  |  |  |
| - Total Moves (0-200) | < 70 | 48 | 82 | Lower Normal Level | 13 | 112 | Upper Normal Level | 34 | 94 | Normal | 38 | 90 | Normal | 44 | 86 | Lower Normal Level | 49 | 80 | Lower Normal Level |
| - Correct Total Moves (0-10) | < 70 | 4 | 96 | Normal | 5 | 102 | Normal | 4 | 96 | Normal | 3 | 90 | Normal | 3 | 92 | Normal | 1 | 80 | Lower Normal Level |
| - Start Time | < 70 | 80 | 110 | Normal | 60 | 102 | Normal | 81 | 110 | Normal | 26 | 92 | Normal | 41 | 96 | Normal | 30 | 92 | Normal |
| - Execution Time | < 70 | 356 | 74 | Borderline | 162 | 106 | Normal | 199 | 100 | Normal | 174 | 104 | Normal | 268 | 84 | Lower Normal Level | 230 | 90 | Normal |
| - Total Time | < 70 | 436 | 76 | Borderline | 222 | 104 | Normal | 280 | 96 | Normal | 200 | 106 | Normal | 309 | 90 | Normal | 260 | 96 | Normal |
| - Time Violations | < 70 | 1 | 86 | Lower Normal Level | 0 | 108 | Normal | 0 | 108 | Normal | 0 | 108 | Normal | 0 | 108 | Normal | 0 | 108 | Normal |
| - Rules Violations | < 70 | 3 | <60 | Deficit | 0 | 104 | Normal | 0 | 104 | Normal | 0 | 104 | Normal | 0 | 106 | Normal | 0 | 106 | Normal |
| **Social Cognition** | | | | | | | | | | | | | | | | | | | |
| Ekman 60 – Faces Test ^12^ | ≤ 37.46 | 46 | 45.16 | 2 | 42 | 41.16 | 1 | 46 | 41.19 | 1 | 45 | 40.19 | 1 | 49 | 44.92 | 2 | 42 | 37.92 | 1 |

**References of cognitive tests**

1. Santangelo G, Siciliano M, Pedone R, et al. Normative data for the Montreal Cognitive Assessment in an Italian population sample. *Neurol Sci.* 2015;36(4):585-591. doi:10.1007/s10072-014-1995-y.

2. Della Sala S, Laiacona M, Spinnler H, Ubezio C. A cancellation test: its reliability in assessing attentional deficits in Alzheimer's disease. *Psychol Med.* 1992;22(4):885-901. doi:10.1017/s0033291700038460.

3. Giovagnoli AR, Del Pesce M, Mascheroni S, Simoncelli M, Laiacona M, Capitani E. Trail making test: normative values from 287 normal adult controls. *Ital J Neurol Sci.* 1996;17(4):305-309. doi:10.1007/BF01997792.

4. Monaco M, Costa A, Caltagirone C, Carlesimo GA. Forward and backward span for verbal and visuo-spatial data: standardization and normative data from an Italian adult population [published correction appears in Neurol Sci. 2015 Feb;36(2):345-7. doi: 10.1007/s10072-014-2019-7]. *Neurol Sci.* 2013;34(5):749-754. doi:10.1007/s10072-012-1130-x.

5. Carlesimo GA, Caltagirone C, Gainotti G. The Mental Deterioration Battery: normative data, diagnostic reliability and qualitative analyses of cognitive impairment. The Group for the Standardization of the Mental Deterioration Battery. *Eur Neurol.* 1996;36(6):378-384. doi:10.1159/000117297.

6. Caffarra P, Vezzadini G, Dieci F, et al. A short version of the Stroop test: Normative data in an Italian population sample. *Nuova Rivista di Neurologia.* 2002; 12:111-115.

7. Spitoni GF, Bevacqua S, Cerini C, et al. Normative Data for the Hayling and Brixton Tests in an Italian Population. *Arch Clin Neuropsychol.* 2018;33(4):466-476. doi:10.1093/arclin/acx072.

8. Costa A, Bagoj E, Monaco M, et al. Standardization and normative data obtained in the Italian population for a new verbal fluency instrument, the phonemic/semantic alternate fluency test. *Neurol Sci.* 2014;35(3):365-372. doi:10.1007/s10072-013-1520-8.

9. Nocentini U, Giordano A, Di Vincenzo S, Panella M, Pasqualetti P. The Symbol Digit Modalities Test - Oral version: Italian normative data. *Funct Neurol.* 2006;21(2):93-96.

10. Saetti MC, Difonzo T, Sirtori MA, et al. *Neuropsychol Trends*. 2021;(29):65-82. doi:10.7358/neur-2021-029-saet.

11. Culbertson CW, Zillmer E. The Tower of London, Drexel University, research version: Examiner’s Manual. North Tonawanda, NY: Multi-Health Systems Inc.; 1999.

12. Dodich A, Cerami C, Canessa N, et al. Emotion recognition from facial expressions: a normative study of the Ekman 60-Faces Test in the Italian population. *Neurol Sci.* 2014;35(7):1015-1021. doi:10.1007/s10072-014-1631-x.
